# Pediatric Palliative Care for Children with Progressive Non-Malignant Diseases

**DOI:** 10.3390/children5020028

**Published:** 2018-02-20

**Authors:** Harold Siden

**Affiliations:** Canuck Place Children’s Hospice, BC Children’s Hospital, University of British Columbia, Vancouver, BC V6H 3N1, Canada; hsiden@cw.bc.ca; Tel.: +1-604-875-2776

**Keywords:** palliative care, palliative medicine, terminal care, hospice care, metabolic diseases, inborn genetic diseases, social support, disease progression, symptom management, emotional support

## Abstract

A substantial number of children cared for by pediatric palliative care physicians have progressive non-malignant conditions. Some elements of their care overlap with care for children with cancer while other elements, especially prognosis and trajectory, have nuanced differences. This article reviews the population, physical-emotional and social concerns, and trajectory.

## 1. Introduction

In industrialized countries the majority of children cared for in Pediatric Palliative Care (PPC) programs have non-malignant diseases other than cancer. This includes a very broad range of conditions affecting the brain, the muscles, the heart and lungs. Infrequently there are infectious and immunologic conditions.

In North America cancer comprises only 30–40% of the cases seen by pediatric palliative care clinical teams. This is a testimony to the overall rarity of childhood cancer and to the overarching success of current treatments. The most common of all the childhood cancers, acute lymphoblastic leukemia, also has the highest 5-year event-free survival rate, at over 90%.

Cancer is widely identified with palliative care in part for historic reasons. The first modern hospice, St. Christopher’s in London, was founded by a medical oncologist, Dame Cicely Saunders. A surgical oncologist, Balfour Mount, coined the term “palliative care”. It was not until some 15 years later that the first hospice for children was developed by Sister Francis Dominica with Helen House, Oxford. Helen, for whom the program is named, indeed had cancer, however it was the central nervous system sequelae of that tumor that made her a candidate for palliative care [1].

This article will describe the broad epidemiology of the non-cancer conditions seen by PPC clinicians, provide insights into aspects of care, and point out areas needing focused research.

## 2. Terminology and Classification

In order to understand the approach to conditions other than cancer followed by PPC teams, it helps to understand terminology and current classification schemes. All of these are continuously evolving as we come to understand the nuances of PPC.

One area of terminology that has led to some confusion is an ongoing discussion over the terms “life-limiting” and “life-threatening”, as different groups use the terms differently [2]. The focus of pediatric palliative care has long been ameliorating symptoms, maintaining good quality of life, and supporting families when a child has a condition that is highly likely to end in a premature death at any time prior to adulthood. When programs, clinicians and researchers use terms such as life-threatening, life-limiting, or any other, they simply need to be explicit about the term they are using and how it is defined. This approach will be more productive than attempting to find the single unifying language, given that all of them are constructs.

The widely cited report of the U.K. charity Together for Short Lives (previously ACT) and the Royal College of Pediatrics and Child Health identified 4 groups of diseases that may receive PPC services [3]. The first of the 4 categories were those diseases where cure was generally possible but for an individual patient failed. Fortunately, the number of curable conditions is growing. The most prominent in this category is Cancer of course (congenital heart disease being a close second). This points out that the conditions seen in the other 3 categories make up the majority of diseases seen in PPC. Category 2 are conditions whereby cure is not possible, but treatments directed at the underlying disease pathophysiology greatly prolong the life-span and enable good quality of life for a long period of time. The classical example is cystic fibrosis. HIV, if full-treatment is available, is another example. In the past few years an increasing number of gene-based metabolic conditions, such as some lysosomal storage diseases, have moved into Category 2. Many of the gene-based metabolic and neurological diseases remain in Category 3. In this category, there is no cure, and no direct treatment for the disease. The focus is on treating manifestations of the disease, such as seizures, inadequate nutrition, secondary osteopenia, etc. The interventions for these conditions namely anti-convulsants, gastrostomy feeding, bisphosphonate infusion, all improve the quality of life and arguably prolong life. They do not, however, stop disease progression. Fortunately, as can now be seen with some storage diseases, and most recently with Spinal Muscular Atrophy, medical advances move diseases from Category 3 to Category 2. Lastly, Category 4 contains neurological conditions secondary to static encephalopathy. In this category we find children who have experienced hypoxic-ischemic brain injury, as well as those with congenital brain dysgenesis. Until we find ways to directly repair injured brains, the focus for care of these individuals is on symptom management and quality of life.

Another lens is to use a simple classification scheme of 7 categories of diseases seen in PPC; breaking them down into: Cancer; Primary CNS conditions; Biochemical/Metabolic diseases; Neuromuscular diseases; Cardiac and Pulmonary conditions; Infectious diseases and Immunologic diseases; Multi-Organ conditions due to chromosomal aneuploidy or gene defects. This is a simplified, practical 7 category system used for internal reporting purposes at Canuck Place Children’s Hospice that has been found useful as a descriptive tool for the estimated 170 different diseases found among the children seen in the program [4]. Others include the system developed by Richard Hain and colleagues, as well as the Complex Chronic Conditions categorization developed by Chris Feudtner and colleagues [5,6]. These categorical systems enable researchers and health services planners to look for trends and evaluate different approaches to treatment.

## 3. Symptom Management

The principles and practice of symptom management with non-cancer conditions are similar to those for cancer, although with some nuanced differences. Attention to these differences may be important [7]. Much of what we know about symptoms in these children derives from our study of 275 children with non-curable, non-cancer conditions. In these children we tracked 7 symptoms: pain, dyspnea, nausea and vomiting, dysomnia, constipation, seizures, and changes in alertness. Strikingly on average, their parents reported 3.2 symptoms of concern for each child [8].

### 3.1. Pain

When discussing symptoms in PPC, attention is immediately paid to pain. Most non-cancer conditions can have pain, but generally not due to the generalized inflammation or space-occupying lesions found in cancer, nor with the marked, ongoing escalation [8,9]. Sources of pain include musculoskeletal pain secondary to weakness, bone and joint conditions, and contractures. Skin and organ pain (due to enlargement) can be found in some genetic metabolic diseases. Pain secondary to procedures and interventions is a problem. Visceral pain, especially gastro-intestinal pain arising all along the alimentary tract can be found in almost all conditions, especially if associated with non-oral feeding, reflux disease and constipation. Neuropathic pain can arise from neuropathy or in association with long-standing nociceptive, inflammatory pain. Lastly, children with central nervous system conditions can also be affected by a “central” pain or neuro-irritability that does not seem to have a nociceptive-inflammatory source [10,11]. This condition is challenging to diagnose and to manage; it is an area of active research.

Treating pain in these children, especially those who are very young or are non-verbal, requires patience, sometimes tenacity, and attention to detail [12]. One must search for readily treatable causes such as hip dysplasia, chronic constipation, or poorly adjusted seating systems [9]. When no cause is found clinicians should pursue a screening approach that takes into account silent conditions, such as renal stones, especially with a young or non-verbal patient. In this circumstance pain is relieved when the underlying cause is directly addressed. The treatment of pain (and many other symptoms described in this article) often requires the input of many disciplines. Medical and surgical interventions are only two components. The involvement of physiotherapists, occupational therapists, and speech-language pathologists is often required to address pain and/or other symptoms.

If no cause is identified, then families and clinicians are faced with “pain-like” behaviors that look like what happens with nociception or inflammation but may be induced by activation of internal central nervous circuits alone. One then undertakes empiric treatment with environmental modifications (e.g., swaddling, massage) and with medications (e.g., gabapentin trial) [13]. A step-wise, trial and error approach using validated assessment tools is needed, along with close communication between clinicians and patients/family members. The nature of this central pain—irritability and approaches to its treatment are areas of active research.

### 3.2. Dysomnia

The second most common symptom in our study was dysomnia. Disturbances of sleep in children can be multi-dimensional and include problems with delayed onset, frequent awakening with fragmented sleep, inadequate sleep duration, and daytime sleepiness (so called “day-night reversal”). For some children many of these features are mixed together. Singly or combined they can pose a major challenge to the child’s health and to the family’s overall wellbeing. They are very common in children with neurological conditions, comprising a large number of the children followed in PPC [14,15]. There are many potential causes that need to be considered in the evaluation including physical health conditions such as upper airway obstruction; poorly-treated pain leading to poor sleep, and environmental factors. Some conditions, such as Rett Syndrome and Mucopolysaccharidosis Type III are well known to have sleep disturbances, probably on a primary neurological basis [16,17,18].

Treatment is highly empirical, and evidence is mostly based on either surveys of current practices (by both clinicians and parents), or on case reports. There is a widespread agreement that environmental and behavioral interventions are worth under-taking. Examples include structured bedtime routines, dark and quiet rooms, avoiding pre-bedtime stimulation with videos. The drawback is that such interventions need to be individualized for each patient’s particular circumstance and therefore it can be time-consuming to find the interventions that work best. Similarly, there needs to be consistency in implementing them. There is evidence, albeit not very strong, for the use of melatonin [14]. Melatonin appears to assist with sleep onset but may lead to a shortened total duration of sleep; using a combination of immediate-acting and long-acting melatonin may avoid this problem. The environmental interventions and melatonin are considered to be safe. Other medications used are a wide variety of drugs with sedation effects—these include anti-histamines, chloral hydrate and benzodiazepines. Chloral hydrate’s only indication is sedation but there are concerns about its long-term use and side effects, especially the effect on the liver. The other medications, as well as a variety of anti-depressants and anti-psychotics, are all sedating but sedation/hypnosis is not their main indication. Specialists however find them useful, and the non-expert prescriber should become familiar with their use and side effects and not hesitate to ask for expert input. Almost every review of dysomnia in children with severe diseases emphasizes the need for family support, especially practical support such as having a night-time nurse or sitter.

### 3.3. Feeding Difficulties and Constipation

In children with non-cancer, life-threatening conditions the 3rd and 4th most common troubling symptoms were feeding difficulties and constipation [8]. The evaluation and treatment of feeding difficulties in children who receive artificial enteral nutrition has been well described. Similarly, there are a number of approaches to treating constipation that can be considered. There are two points worth noting: one is that treating feeding intolerance requires a step-wise, patient approach. Similarly, treating constipation involves more than simply prescribing a stool softener and assuming the problem is resolved. It requires ongoing assessment of the situation. The second is that in some circumstances feeding intolerance, especially intolerance of elemental formulas and electrolyte solutions in the absence of signs of malabsorption, may be a harbinger of terminal decline. This has been reported but not widely studied [19].

Due to the preponderance of neurological disorders found in children with non-cancer diagnoses, artificial enteral nutrition is a common feeding approach. Not only can these children have difficulties secondary to reflux, delayed gastric emptying and constipation, but their parents also report a higher prevalence of pain and respiratory problems. Pain may be a component of the gastrointestinal symptoms, or another manifestation of the overall disease condition. Respiratory difficulties again may be due to reflux, perhaps exacerbated by the addition of a feeding tube, or it may be se secondary to inadequate airway protection and ongoing aspiration of saliva and bronchial secretions. In some centers this may be an indication for tracheostomy, but it has not been the practice at our institution.

### 3.4. Dyspnea

Dyspnea, the sensation of breathlessness, was also found in our longitudinal study. Dyspnea is a symptom best studied in the context of adult cancers, especially those involving the lungs, the airway, or the muscle system. While the sensation is well described, there are few commonly used tools to assess it, and a minimal literature on treatment—the literature emphasizes the use of airflow across the face, and opioids to alter the CO_2_ ventilation threshold so that there is reduced stimulus towards compensatory hyperventilation.

Dyspnea is primarily understood in the context of adult cancer and cardio-respiratory medicine. Scales have been developed in children, such as the Dalhousie Dyspnea Scales [20]. These scales were developed with verbal, cooperative children over 8 years of age, in situations such as severe asthma and cystic fibrosis. There has not been a similar scale developed for non-verbal, non-cooperating children. Overall there is a paucity of research [21]. Clinically there is a challenge in determining whether a child with severe neurological impairment, from whatever cause, is experiencing dyspnea. Clinicians may assume that the child with tachypnea, use of accessory respiratory muscles, decreased oxygen saturation and other sings of respiratory distress are experiencing dyspnea. They may empirically utilize dyspnea plans with interventions such as airflow, opioids and benzodiazepines with the expectation of benefit.

### 3.5. Neurological Symptoms

There are other symptoms of concern that must be addressed in children with non-cancer, life-threatening illnesses, especially neurological symptoms [22]. First among these are seizures. Closely working with the child’s neurologist will be the best step towards determining an optimal plan for seizure control. In some cases, full control cannot be achieved. Developing both an optimal target and a rescue plan for prolonged or frequent seizures is critical.

Along these lines dystonia is a challenging symptom to contend with [23]. The first step in treating dystonia is to recognize that it is distinct from seizures, although to the uninformed observer, and especially with a non-verbal patient, the uncoordinated movements may suggest a convulsion. Children may also have co-existing dystonia and seizure disorder, further complicating the picture. Diagnostic evaluation needs to include an EEG to evaluate and delineate seizure-related movements and to treat those. While treatment of dystonia may involve muscle relaxants such as baclofen, parasympathetic inhibitors (trihexyphenidyl), benzodiazepines and dopamine agonists are often tried; these all depend on knowing the etiology of the condition.

Two other symptoms warrant consideration as parents may raise them, although very little is actually known about them. One is lack of arousal or easy fatigability, while the other is temperature dysregulation (cold or hot extremities). These two symptoms have appeared in reports by parents as troubling to them [24]. The lack of arousal is suggested when a parent notes that the child “is no longer him/herself” at times, or less interactive than usual. While the children are in many cases very impaired in language, parents and other caregivers who know the child well learn the individual signals that indicate preferences and emotions. When these are absent, it is clear to the family and close caregivers that something is different. There may be many causes to be investigated, including the introduction of new medications, neurological changes—for example more seizures, or fatigue due to lack of good sleep. More ominously, it may indicate progression of an underlying disease, especially for metabolic conditions related to energy or storage diseases. Some clinicians have speculated that a change in arousal might be related to a psychiatric depression; our current state of understanding the emotional life of a child with neurological impairment is very limited, so we must consider these speculations with reservation.

Temperature dysregulation is also reported by parents, especially in reference to cold and hot extremities. This is also an area that has not received much attention in the literature. One reason that hands and feet may be cold is that the child may lack large muscle mass due to inactivity, resulting in decreased thermal production. When the extremities alternate between being hot and cold, then centrally based autonomic dysfunction may be the etiology. We are learning more about the role that the autonomic nervous system plays in children and this may lead to useful interventions [25]. In the short term, simple environmental measures are useful paying attention to cold and hot hands and feet and changing clothing accordingly.

## 4. Emotional Support

It is fundamental that emotional support for the affected child is a responsibility of pediatric palliative care. In this regard there is no difference between children experiencing cancer and those with non-cancer diagnoses. In both situations the range of services must be broad and robust. There needs to be capacity to provide many different modalities adjusted to age, developmental capacity, and child/youth preferences. Modalities employed include play-based therapy, art therapy, music therapy, and standard talk-based approaches such as cognitive-behavior therapy.

Just because a child is very young or non-verbal it is wrong to assume that they are not experiencing feelings that need to be addressed. Many parents of non-verbal children provide consistent descriptions of their child’s understanding that usually exceeds what a brief observation in a clinic will demonstrate to a clinician. Prolonged interaction and observation of a child by nurses, physical and occupational therapists, and others generally confirms the parents’ assessment. Therefore, taking the lead from parents and developing a strategy to address a child’s depression, anxiety, and fears, regardless of perceived developmental stage is important.

## 5. Social Issues

It is here that we consider the family beyond the affected child; parents and siblings, and in many cases, grandparents, aunts and uncles and other extended members. Foremost is addressing their questions that may focus on prognosis; this will be covered further on under the Trajectory section. Social support for family includes both care for their emotional needs—anxiety, depression, guilt—but also the practical issues of daily life that are complicated by having a child with a life-threatening condition.

Some observations about how non-cancer conditions can be different are warranted here. One is that while “cancer” is not a single diagnosis, the care of children with cancer is often provided by a unified team at a single center. Parents then benefit from services available at a single site under the banner of cancer care; this may include mental health services for themselves and for siblings, family support through activities such as camps, and a communal experience with other families encountered in the clinic and hospital. Simply having a recognizable condition such as cancer, as difficult as that is, may create a framework for social understanding.

Non-cancer diagnoses number in the hundreds and cover a wide variety of organ systems [26]. There is no single team or clinic that may follow these children. There is often not a support system for parents or siblings provided in an ongoing, organized fashion, and families may feel highly isolated. As most of these conditions are very rare, families may struggle to explain to others what exactly is going on in their lives.

A particular challenge for families are one of a kind diagnoses where there are no other children with a similar condition, and similarly where a child lacks a diagnosis. In both cases families are left without information, increasing the uncertainty. New techniques in genome analysis, such as Whole Exome Sequencing and Whole Genome Sequencing are now available at both the clinical and research level. However, not all gene-based conditions can be identified as due to a single gene mutation. In some cases, there are multiple gene interactions that lead to a phenotype. Understanding how the protein product of a gene creates the condition is a necessary piece of information. Recent studies suggest that families’ value a “diagnosis” even when it does not lead to any treatment [27].

Regardless of having the gene explanation, families with children whose condition is either very rare or non-diagnosed can feel isolated. This can occur both in their family and community as well as with interactions in the health system. It is not unusual for families to report confusion by health care providers and sometimes reluctance to provide care. It is important for clinicians to understand that in the absence of a life-prolonging or curative treatment, the focus on symptom management will rely on basic principles that are similar across conditions, until we learn otherwise.

Because of their expertise in symptom management, familiarity with rare and undiagnosed conditions, and family-centered approach, pediatric palliative care teams can be important to families, sometimes akin to a “medical home” for families if they are resourced accordingly. If not that well resourced, they can still be valuable as consultants to primary teams caring for these children.

## 6. Trajectory

In the model we teach to trainees and use day-to-day in practice, following the sequence outlined as Physical, Emotional (child), Social (family), Spiritual (religion and meaning), and Trajectory, the last item keying into advance care planning. Trajectory is a topic that uncovers child and family goals, hopes and concerns. Based on these elements we then develop alternative pathways using a “what if” approach. This is an iterative process.

As described in the previous sections, non-malignant conditions are often hard to characterize as they are rare, not well studied, and with a resulting low level of evidence. As many of these conditions have a genetic basis, the nature of the gene defect, balancing and unbalancing genetic factors, and interaction with the environment all play a role in phenotype and therefore clinical course. These factors all combine to make disease course and prognostication very difficult. A high degree of clinical expertise, experience, careful communication, and a healthy dose of skepticism are all needed.

**Figure 1:**
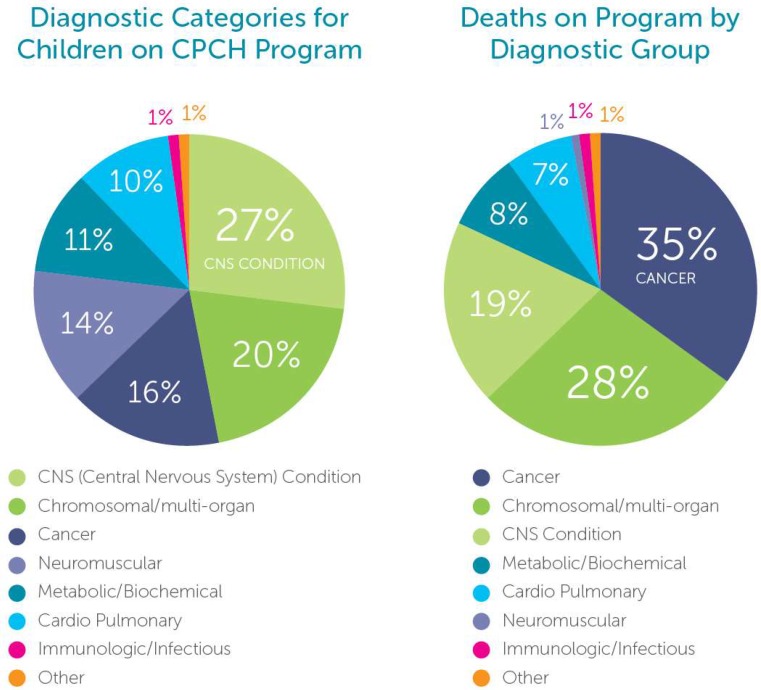
Children on Program and Deaths on Program Canuck Place Children’s Hospice.

Figure 1 gives insight into an interesting finding; the majority of children followed at any given time on the Canuck Place program have non-cancer diseases, whereas mortality rates show a high incidence of cancer deaths.

**Figure 2:**
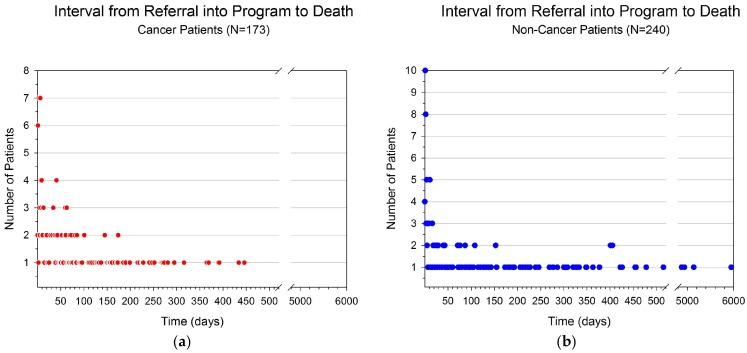
(**a**) Intervals from referral to palliative care until death for cancer patients; (**b**) Intervals from referral to palliative care until death for patients with non-cancer conditions.

This pattern suggests that there are differences in the way that, and the timing of, engagement of children and their families with PPC services at least in one well-established program. Similar data is found when examining timing of death in relationship to referral, broken down by disease category as shown in Figure 2. Children with cancer may die sooner after referral as curative attempts continue well into the disease course. For many patients with non-cancer, non-curable conditions, clinicians and families are seeking hospice, medical respite, and palliative care support earlier in the disease, and therefore constitute a larger proportion of patients on program at any given time. This is one hypothesis to explain the differences. In addition, the services accessed for patients and families with cancer as compared to a non-cancer diagnosis may differ. Care intensity may be high in children and families living with cancer, although for a shorter period of time. In contrast, children with non-cancer diagnoses, especially those with static encephalopathy (Together for Short Lives Category 4), received care intermittently but over longer-time periods [28].

As was shown in Figure 2 the unpredictable pattern of time from referral to death, observed in several studies, confirms that the best approach is to prepare families, and oneself, both for sudden terminal events and simultaneously for a protracted course. Preparing families involves both education and careful planning. One mother aptly describes this as “only a (parent in a pediatric palliative care program) meets their kindergarten principal and funeral director in the same week”.

In addition to discussing advance care planning and advance directives with families as ongoing discussion, it is also advisable to explore scenarios. Completing paperwork, for example a letter to be used in the Emergency Room, is also advisable so that one is prepared for the unexpected.

The pediatric palliative care program at Canuck Place does not require an advance directive document or Do Not Attempt Resuscitation order. It is practice however to have advance care planning discussions with families on an annual basis, at minimum. In these discussions it may be sufficient to simply review the relevant concepts and terminology that families may encounter (e.g., what is a “Code Blue”), without requiring any specific decision-making. As we found in a retrospective review, this kind of conversation supports family decision-making, and one result is that even among the families who self-described as choosing all resuscitation measures, at the time of their child’s death almost none asked for intervention.

## 7. Future Directions

As the field of pediatric palliative care matures more attention is being paid to the non-malignant conditions in research and knowledge translation. An understanding that cross-communication among several fields and subspecialties in medicine and other disciplines needs to occur in order to develop a more comprehensive (holistic) understanding of how these conditions are experienced by affected children and their families, and in turn to design interventions that support quality of life.

Immediate targets include epidemiological and health services research studies to better delineate the population of interest and their service use; clinical intervention studies of physical symptoms, children’s emotional wellbeing, and family psycho-social health; and basic physiology and pharmacology studies to enhance our understanding of symptoms and symptom treatment.

Children and their families with non-malignant conditions are a substantial proportion of the work of pediatric palliative care and will potentially increase in number in the future with advances in life-prolonging treatments. This is an area that deserves our attention especially for on-going research.

## References

[1] Worswick J. (2000). A House Called Helen: The Development of Hospice Care for Children.

[2] Spicer S., Macdonald M., Davies D., Vadeboncoeur C., Siden H. (2015). Introducing a Lexicon of Terms for Pediatric Palliative Care. Paediatr. Child Health.

[3] ACT (The Association for Children’s Palliative Care) (1997). RCPCH A Guide to the Development of Children’s Palliative Care Services: Report of the Joint Working Party of the Association for Children with Life-Threatening or Terminal Conditions and Their Families (ACT) and the Royal College of Paediatrics and Child Health (RCPCH).

[4] Chavoshi N., Miller T., Siden H. (2013). Resource utilization among individuals dying of pediatric life-threatening diseases. J. Palliat. Med..

[5] Bergstraesser E., Hain R.D., Pereira J.L. (2013). The development of an instrument that can identify children with palliative care needs: The Paediatric Palliative Screening Scale (PaPaS Scale): A qualitative study approach. BMC Palliat. Care.

[6] Feudtner C., Christakis D.A., Connell F.A. (2000). Pediatric Deaths Attributable to Complex Chronic Conditions: A Population-Based Study of Washington State, 1980–1997. Pediatrics.

[7] Malcolm C., Hain R., Gibson F., Adams S., Anderson G., Forbat L. (2012). Challenging symptoms in children with rare life-limiting conditions: Findings from a prospective diary and interview study with families. Acta Paediatr..

[8] Steele R., Siden H., Cadell S., Davies B., Andrews G., Feichtinger L., Singh M. (2014). Charting the territory: Symptoms and functional assessment in children with progressive, non-curable conditions. Arch. Dis. Child..

[9] Siden H., Oberlander T. (2008). Pain management for children with a developmental disability in a primary care setting. Pain in Children: A Practical Guide for Primary Care.

[10] Oberlander T.F., O’Donnell M.E. (2001). Beliefs about pain among professionals working with children with significant neurologic impairment. Dev. Med. Child Neurol..

[11] Oberlander T.F., Symons F., Van Dongen K., Abu-Saad H.H. (2003). Pain in Individuals with Developmental Disabilities: Challenges for the Future. Prog. Pain Res. Manag..

[12] Siden H.B., Carleton B.C., Oberlander T.F. (2013). Physician variability in treating pain and irritability of unknown origin in children with severe neurological impairment. Pain Res. Manag. J. Can. Pain Soc. J. Soc. Can. Pour Trait. Douleur.

[13] Hauer J.M., Wical B.S., Charnas L. (2007). Gabapentin Successfully Manages Chronic Unexplained Irritability in Children with Severe Neurologic Impairment. Pediatrics.

[14] Braam W., Didden R., Smits M., Curfs L. (2008). Melatonin treatment in individuals with intellectual disability and chronic insomnia: A randomized placebo-controlled study. J. Intellect. Disabil. Res..

[15] Jan J.E., Freeman R.D. (2004). Melatonin therapy for circadian rhythm sleep disorders in children with multiple disabilities: What have we learned in the last decade?. Dev. Med. Child Neurol..

[16] Piazza C.C., Fisher W., Kiesewetter K., Bowman L., Moser H. (1990). Aberrant sleep patterns in children with the Rett syndrome. Brain Dev..

[17] Mariotti P., Della Marca G., Iuvone L., Vernacotola S., Ricci R., Mennuni G.F., Mazza S. (2003). Sleep disorders in Sanfilippo syndrome: A polygraphic study. Clin. EEG Electroencephalogr..

[18] Zucconi M., Bruni O. (2001). Sleep disorders in children with neurologic diseases. Semin. Pediatr. Neurol..

[19] Siden H., Tucker T., Derman S., Cox K., Soon G.S., Hartnett C., Straatman L. (2009). Pediatric enteral feeding intolerance: A new prognosticator for children with life-limiting illness?. J. Palliat. Care.

[20] Pianosi P., Smith C.P., Almudevar A., McGrath P.J. (2006). Dalhousie dyspnea scales: Pictorial scales to measure dyspnea during induced bronchoconstriction. Pediatr. Pulmonol..

[21] Craig F., Henderson E.M., Bluebond-Langner M. (2015). Management of respiratory symptoms in paediatric palliative care. Curr. Opin. Support. Palliat. Care.

[22] Rasmussen L.A., Gregoire M.-C. (2015). Challenging neurological symptoms in paediatric palliative care: An approach to symptom evaluation and management in children with neurological impairment. Paediatr. Child Health.

[23] Allen N.M., Lin J.-P., Lynch T., King M.D. (2014). Status dystonicus: A practice guide. Dev. Med. Child Neurol..

[24] Hunt A.M. (1990). A Survey of Signs, Symptoms and Symptom Control in 30 Terminally Ill Children. Dev. Med. Child Neurol..

[25] Rees C.A. (2014). Lost among the trees? The autonomic nervous system and paediatrics. Arch. Dis. Child..

[26] Hain R., Devins M., Hastings R., Noyes J. (2013). Paediatric palliative care: Development and pilot study of a “Directory” of life-limiting conditions. BMC Palliat. Care.

[27] Makela N.L., Birch P.H., Friedman J.M., Marra C.A. (2009). Parental perceived value of a diagnosis for intellectual disability (ID): A qualitative comparison of families with and without a diagnosis for their child’s ID. Am. J. Med. Genet. A.

[28] Bender H.U., Riester M.B., Borasio G.D., Führer M. (2017). “Let’s Bring Her Home First.” Patient Characteristics and Place of Death in Specialized Pediatric Palliative Home Care. J. Pain Symptom Manag..

